# AS1411-conjugated gold nanospheres and their potential for breast cancer therapy

**DOI:** 10.18632/oncotarget.4207

**Published:** 2015-06-03

**Authors:** Mohammad T. Malik, Martin G. O'Toole, Lavona K. Casson, Shelia D. Thomas, Gina T. Bardi, Elsa Merit Reyes-Reyes, Chin K. Ng, Kyung A. Kang, Paula J. Bates

**Affiliations:** ^1^ Departments of Medicine, University of Louisville, Louisville, Kentucky, USA; ^2^ Departments of Bioengineering, University of Louisville, Louisville, Kentucky, USA; ^3^ Departments of Radiology, University of Louisville, Louisville, Kentucky, USA; ^4^ Departments of Chemical Engineering, University of Louisville, Louisville, Kentucky, USA; ^5^ Departments of the Molecular Targets Group of the James Graham Brown Cancer Center, University of Louisville, Louisville, Kentucky, USA

**Keywords:** targeted therapy, nanomedicine, triple negative breast cancer, G-quadruplex, nanoparticles

## Abstract

AS1411 is a quadruplex-forming DNA oligonucleotide that functions as an aptamer to target nucleolin, a protein present on the surface of cancer cells. Clinical trials of AS1411 have indicated it is well tolerated with evidence of therapeutic activity, but improved pharmacology and potency may be required for optimal efficacy. In this report, we describe how conjugating AS1411 to 5 nm gold nanospheres influences its activities *in vitro* and *in vivo*. We find that the AS1411-linked gold nanospheres (AS1411-GNS) are stable in aqueous and serum-containing solutions. Compared to unconjugated AS1411 or GNS linked to control oligonucleotides, AS1411-GNS have superior cellular uptake and markedly increased antiproliferative/cytotoxic effects. Similar to AS1411, AS1411-GNS show selectivity for cancer cells compared to non-malignant cells. In a mouse model of breast cancer, systemic administration of AS1411-GNS could completely inhibit tumor growth with no signs of toxicity. These results suggest AS1411-GNS are promising candidates for clinical translation.

## INTRODUCTION

Our group previously discovered that certain synthetic G-rich DNA oligonucleotides can inhibit the growth of cancer cells without affecting non-malignant cells and this was related to their ability to form G-quadruplex structures that can bind as aptamers to nucleolin protein [1, 2]. In normal cells, nucleolin is localized primarily in the nucleus (in nucleoli), but in cancer cells, it is also present in the cytoplasm and on the cell surface [2–6]. Cell surface nucleolin can mediate the uptake and signaling of various growth factors that help cancers grow, while cytoplasmic nucleolin increases levels of anti-apoptotic mRNAs and miRNAs that help cancer cells survive [2–10]. Thus, the non-nuclear forms of nucleolin are highly cancer-selective targets because they are found preferentially in cancer cells compared to normal cells *and* they have functions that are essential for cancer cell survival but dispensable in normal cells. Accordingly, some molecules that bind to nucleolin—e.g. aptamers [2], peptides [11], and antibodies [6]—have been found to specifically accumulate inside cancer cells and/or to selectively kill cancer cells. One of the nucleolin-binding G-rich oligonucleotides that our group developed, which is commonly known as AS1411 (formerly AGRO100 and now named ACT-GRO-777), was the first nucleolin-targeted agent (and the first anticancer aptamer) to reach human clinical trials. Phase 1 and 2 clinical trials in more than 100 patients with metastatic or advanced cancers have shown that AS1411 has an excellent safety profile and evidence of clinical activity, with many examples of disease stabilization and several cases of long-lasting objective responses [2, 12–14]. However, despite remarkable results in a few patients, the overall rate of response has been low, possibly because AS1411 has less than optimal pharmacology (it is rapidly cleared from the body) and relatively low potency (micromolar concentrations are typically required to induce cancer cell death). We hypothesized that attaching AS1411 to gold nanospheres would increase its accumulation in cancer cells and enhance its antitumor efficacy *in vivo*. In this paper, we describe the physical properties and biological activities of AS1411 conjugated to 5 nm solid gold nanospheres. Consistent with our hypothesis, we report that these AS1411-conjugated gold nanospheres (AS1411-GNS) have significantly improved cancer-targeting activities compared to unconjugated AS1411 or control GNS.

## RESULTS

### Physicochemical properties of nanoparticles

**Figure 1:**
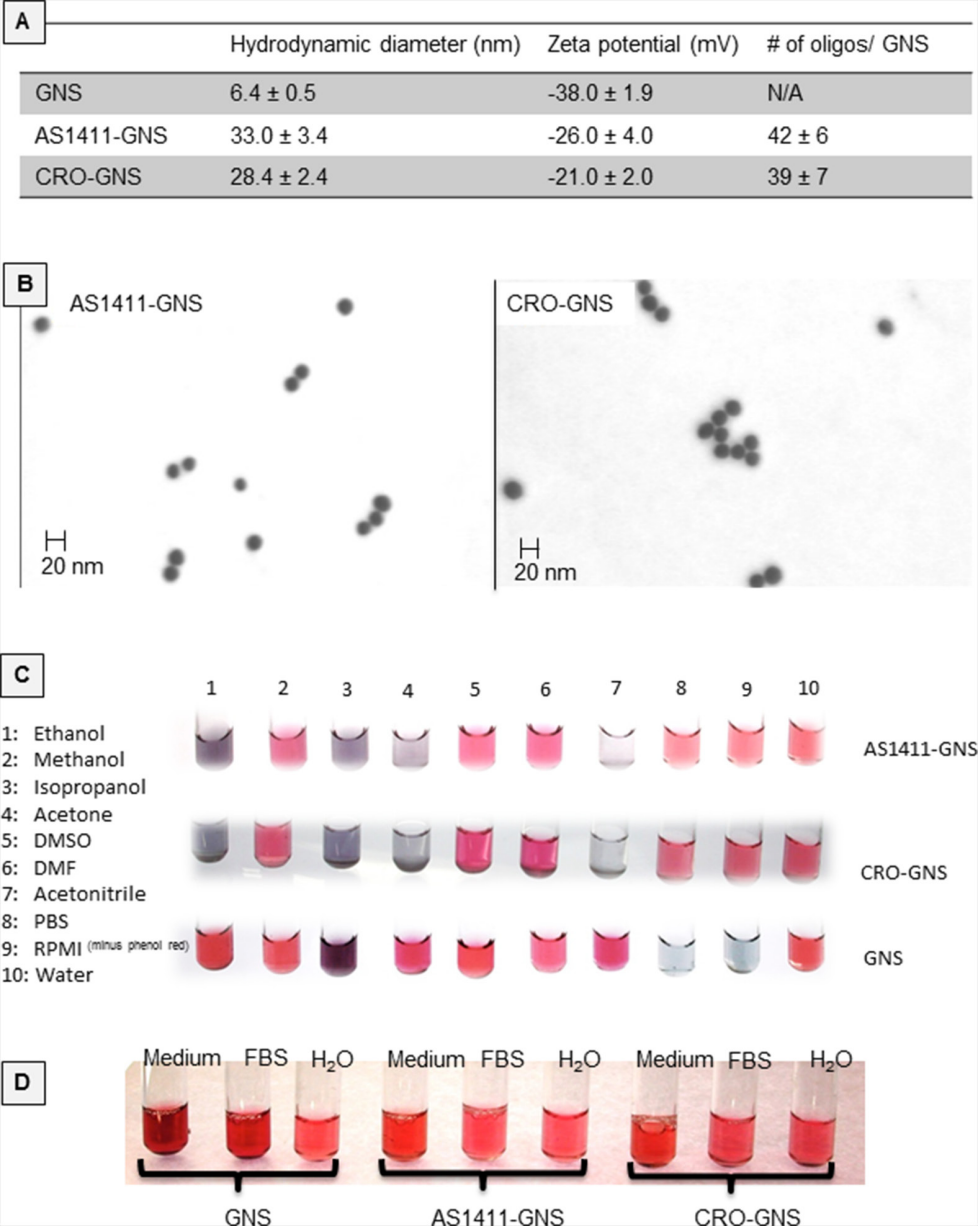
Physicochemical properties of nanospheres **A.** Physical characteristics of oligonucleotide-conjugated GNS compared to citrate-capped GNS. **B.** Oligonucleotide-conjugated GNS imaged by scanning transmission electron microscopy (STEM). **C.** Aliquots of conjugated or unconjugated gold nanospheres were incubated with various solvents for 5 days at room temperature and the color was recorded. Red/pink color was considered indicative of well-dispersed particles (i.e. stability), whereas purple/blue to gray/colorless was considered to indicate increasing degrees of aggregation (i.e. instability). **D.** Various GNSs incubated for 7 days in water, fetal bovine serum (FBS), or complete cell culture medium (RPMI without phenol red + 10% FBS).

Oligonucleotide functionalized gold nanospheres were prepared as described in the Methods section and the resultant complexes were characterized for hydrodynamic diameter, zeta potential, and oligonucleotide density, as shown in Figure 1A. Scanning transmission electron microscopy (STEM, Figure 1B) further confirmed the size of the oligonucleotide-GNS. To assess the stability of nanoparticle suspensions in various solvents, color changes were monitored following incubation for 5 days at room temperature (Figure 1C). Both AS1411-GNS and CRO-GNS remained dispersed (indicated by red or pink color) in water, phosphate buffered saline (PBS), cell culture medium (RPMI without phenol red), methanol, DMSO, and DMF, but aggregated (indicated by gray or clear color) in acetonitrile, ethanol, acetone, and isopropanol. By contrast, the unconjugated (citrate-capped) GNS were more stable in some organic solvents, but aggregated in the high salt aqueous solutions, such as PBS and RPMI (Figure 1C). There was also no evidence of aggregation for AS1411-GNS or CRO-GNS after incubation for 7 days in the presence of fetal bovine serum (FBS) or complete cell culture medium (RPMI without phenol red + 10% FBS), whereas the unconjugated GNS displayed some color changes, as illustrated in Figure 1D. The data shown in Figure 1 demonstrate that the AS1411-GNS and CRO-GNS have comparable physicochemical properties, and also indicate they have characteristics (e.g. size and stability) suitable for development as a nanomedicine.

### Cellular uptake

**Figure 2:**
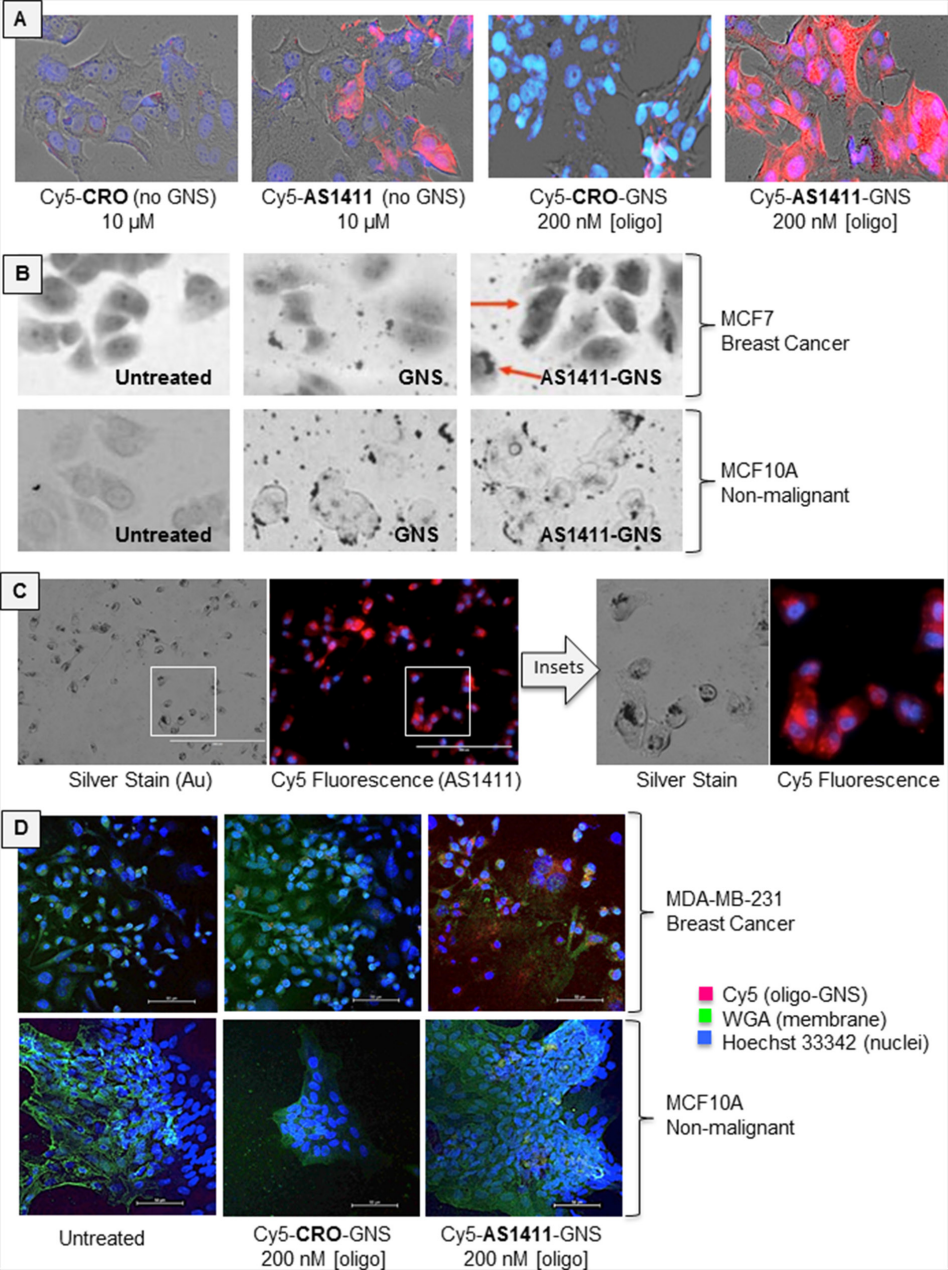
Cellular uptake **A.** MCF7 breast cancer cells were incubated with fluorescent Cy5-linked oligonucleotides (AS1411 aptamer or CRO control), or with Cy5-oligonucleotides attached to 5 nm GNS. Micrographs show brightfield images overlaid with Cy5 fluorescence (red) and DAPI staining (cell nuclei, blue) after 72 h incubation. **B.** Silver-enhanced staining in MCF7 breast cancer cells and MCF10A non-malignant breast epithelial cells. Note that GNS accumulate in the perinuclear region of MCF7 cancer cells (red arrows) only when attached to AS1411, whereas MCF10A non-malignant cells display surface binding of GNS and AS1411-GNS, but very little internalization. **C.** Comparison of silver staining and Cy5 fluorescence in the same sample of MCF7 cells, indicating substantial co-localization. **D.** MDA-MB-231 breast cancer cells and MCF10A cells assessed by confocal microscopy. Micrographs show overlay of Cy5 fluorescence (red), nuclear stain (blue) and WGA stain (green).

We utilized a number of methods to assess AS1411-GNS accumulation in breast cancer cells (MCF-7 and MDA-MB-231) and non-malignant breast epithelial cells (MCF10A). In the studies shown in Figure 2A, we used fluorescently (Cy5) labeled oligonucleotides or oligonucleotide-GNS conjugates and visualized cellular uptake by fluorescence microscopy. In accord with our previous work [15], we observed that AS1411 accumulates in cancer cells at higher levels than the control oligonucleotide (CRO). Moreover, we found that the internalization of AS1411-GNS in MCF-7 breast cancer cells was greatly increased compared with either AS1411 alone or with CRO-GNS (GNS decorated with a control DNA). Notably, uptake of AS1411-GNS was much greater than unconjugated AS1411 even though the aptamer concentration used for the former was much lower (to reflect the differences in their antiproliferative effects). Next, in order to confirm that the gold component of AS1411-GNS is also delivered to the inside of the cancer cells, we used silver-enhanced staining to visualize the localization of the gold particles. With this technique, the GNS catalyze the deposition of silver on their surface, effectively increasing their size and allowing detection by light microscopy. As shown in Figure 2B, there is some background silver staining of proteins in the cell (see “untreated” samples), but when treated with untargeted GNS or AS1411-linked GNS, additional staining is observed. For GNS in MCF-7 cancer cells, and for both GNS and AS1411-GNS in non-malignant MCF10A cells, the silver-enhanced GNS staining is observed mostly outside the cell or associated with the cell surface. Only for AS1411-GNS in cancer cells, was a high concentration of GNS staining observed *inside* the cell, where pronounced perinuclear accumulation was apparent (Figure 2B, arrows). When we compared fluorescence and silver staining in the same sample, substantial co-localization of the fluorophore (attached to AS1411) with the silver aggregates (indicating the GNS) was observed (Figure 2C), suggesting that the Cy5-AS1411-GNS particles remain intact inside the cell. Finally, to verify our findings in a different breast cancer cell line, we visualized the localization of Cy5-labeled oligonucleotide-GNS complexes in MDA-MB-231 breast cancer cells and MCF10A non-malignant cells by confocal microscopy. As a putative plasma membrane marker to delineate cell boundaries in these experiments, we co-stained cells with wheat germ agglutinin (WGA), a lectin that binds to sialic acid and *N*-acetylglucosaminyl residues found predominantly on the plasma membrane in most mammalian cells (though we noted substantial intracellular fluorescence in cancer cells). Again, we observed that AS1411-GNS had higher accumulation in the cancer cells (MDA-MB-231) compared with non-malignant cells (MCF10A), and that cancer cell uptake of AS1411-GNS was much higher than the control (CRO-GNS). Taken together, the results in Figure 2 establish that the AS1411-GNS are efficiently and selectively internalized by breast cancer cells. These data also illustrate that attaching AS1411 to GNS dramatically enhances accumulation in breast cancer cells compared to either component alone.

### Antiproliferative activity

**Figure 3:**
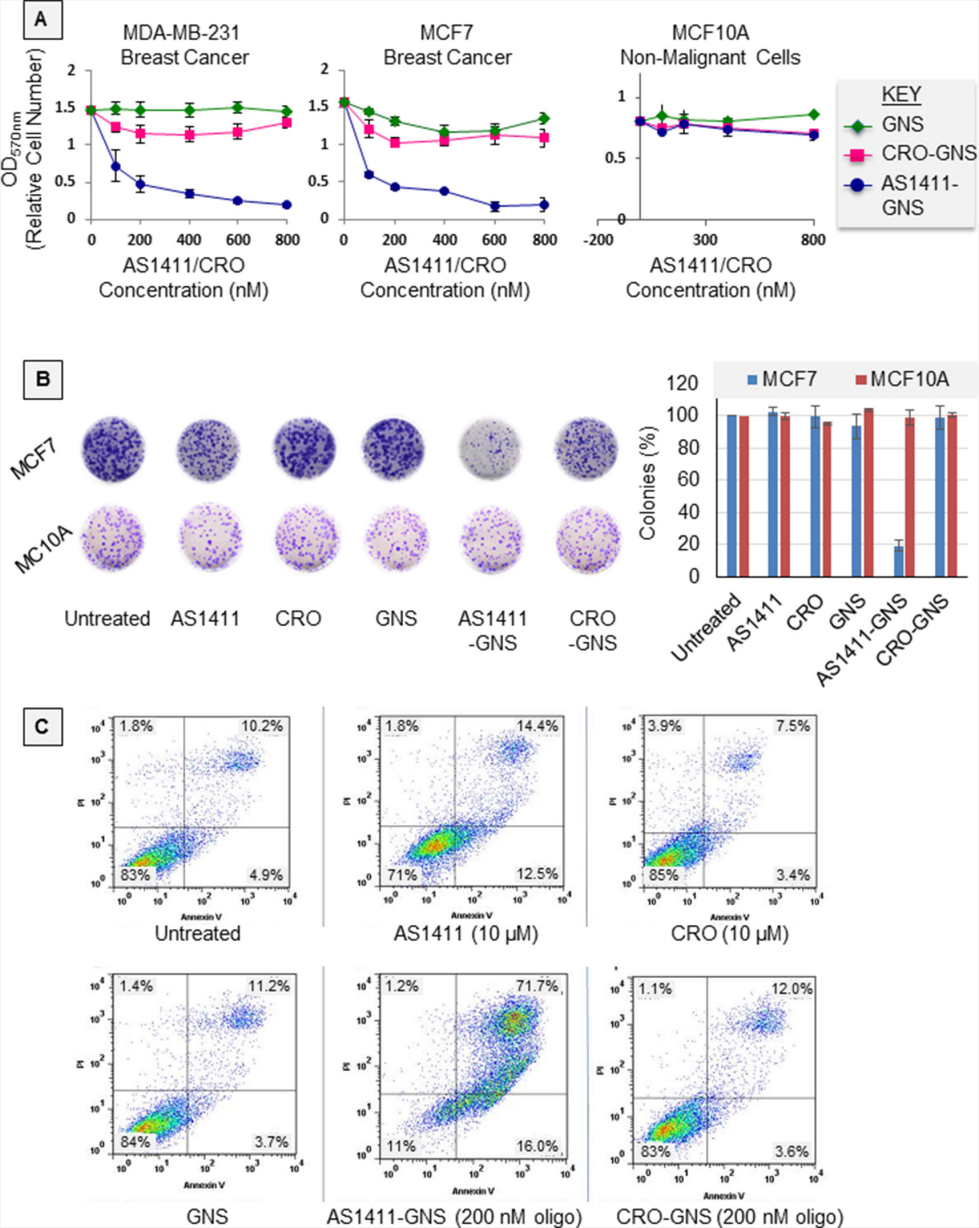
Antiproliferative activity **A.** Graphs showing the effect of various concentrations of AS1411-GNS, or CRO-GNS (control), or an equivalent amount of unconjugated 5 nm GNS on the growth of breast cancer cell lines (MCF7 and MDA-MB-231) and non-malignant breast epithelial cells (MCF10A). Cells were incubated with nanospheres for 72 h and proliferation was measured by MTT assay. Data points represent the mean ± standard error from three independent experiments. **B.** MCF7 breast cancer cells or non-malignant MCF10A breast epithelial cells were incubated for 10 days with equivalent amounts of gold nanospheres (GNS) or GNS attached to either AS1411 aptamer or to CRO control oligonucleotide (final AS1411 or CRO concentration was 200 nM). Image shows representative samples after fixing and staining (left) and a graph indicating the number of colonies with ≥ 50 cells, relative to untreated samples (right, bars indicate the mean and standard error from three independent experiments). **C.** MDA-MB-231 breast cancer cells were treated as indicated in the figure. After 72 h treatment, cell death was determined by flow cytometric analyses of cells stained with FITC Annexin V and propidium iodide (PI). The percentage of cells is shown in each quadrant and indicates live cells (annexin V-negative/PI-negative, bottom left quadrant) compared to dead or dying cells (annexin V-positive and/or PI-positive).

Colorimetric MTT assays were used to test how AS1411-GNS affects the proliferation of breast cancer cells (MDA-MB-231, MCF-7) and non-malignant breast epithelial cells (MCF10A). Figure 3A shows the response of cells incubated with increasing concentrations of AS1411-GNS or equivalent amounts of control nanospheres for 72 h. We found that unconjugated GNS and GNS conjugated to control aptamer CRO had no significant effect on the proliferation of any of the cell lines tested, whereas AS1411-GNS had profound antiproliferative effects on MDA-MB-231 and MCF-7 breast cancer cells. In these cell lines, the GI_50_ (concentration required to inhibit cell growth by 50%) for AS1411-GNS was equivalent to < 100 nM AS1411, which is about 20-fold less than reported for AS1411 alone [10, 16]. Importantly, like AS1411 [10], AS1411-GNS had no major inhibitory effects on the proliferation of the non-malignant MCF10A cells. The impact of AS1411-GNS on breast cancer cell survival and cell death was also tested. We observed that AS1411-GNS could inhibit MCF-7 cell survival in clonogenic assay by more than 80% at 200 nM, whereas neither AS1411 alone nor control GNS had any significant effect under these conditions (Figure 3B). When MDA-MB-231 cell death was analyzed by flow cytometry after staining cells with annexin V/propidium iodide (PI), we observed a dramatic increase in cell death with AS1411-GNS (88% cell death at 200 nM) compared to AS1411 (27% cell death at 10 μM), as shown in Figure 3C. Cells treated with CRO, GNS, or CRO-GNS had levels of cell death similar to the baseline level observed in untreated cells (about 15%). For both AS1411 and AS1411-GNS, cell death was characterized by an increase in both apoptotic cells (Annexin V-positive/PI-negative) and necrosis (Annexin V-positive/PI-positive cells). The combination of results illustrated in Figure 3 indicate that attaching AS1411 to GNS leads to a remarkable increase in potency of the aptamer in terms of its antiproliferative activity and cytotoxicity, while retaining the same cancer-selectivity.

### *In vivo* studies

**Figure 4:**
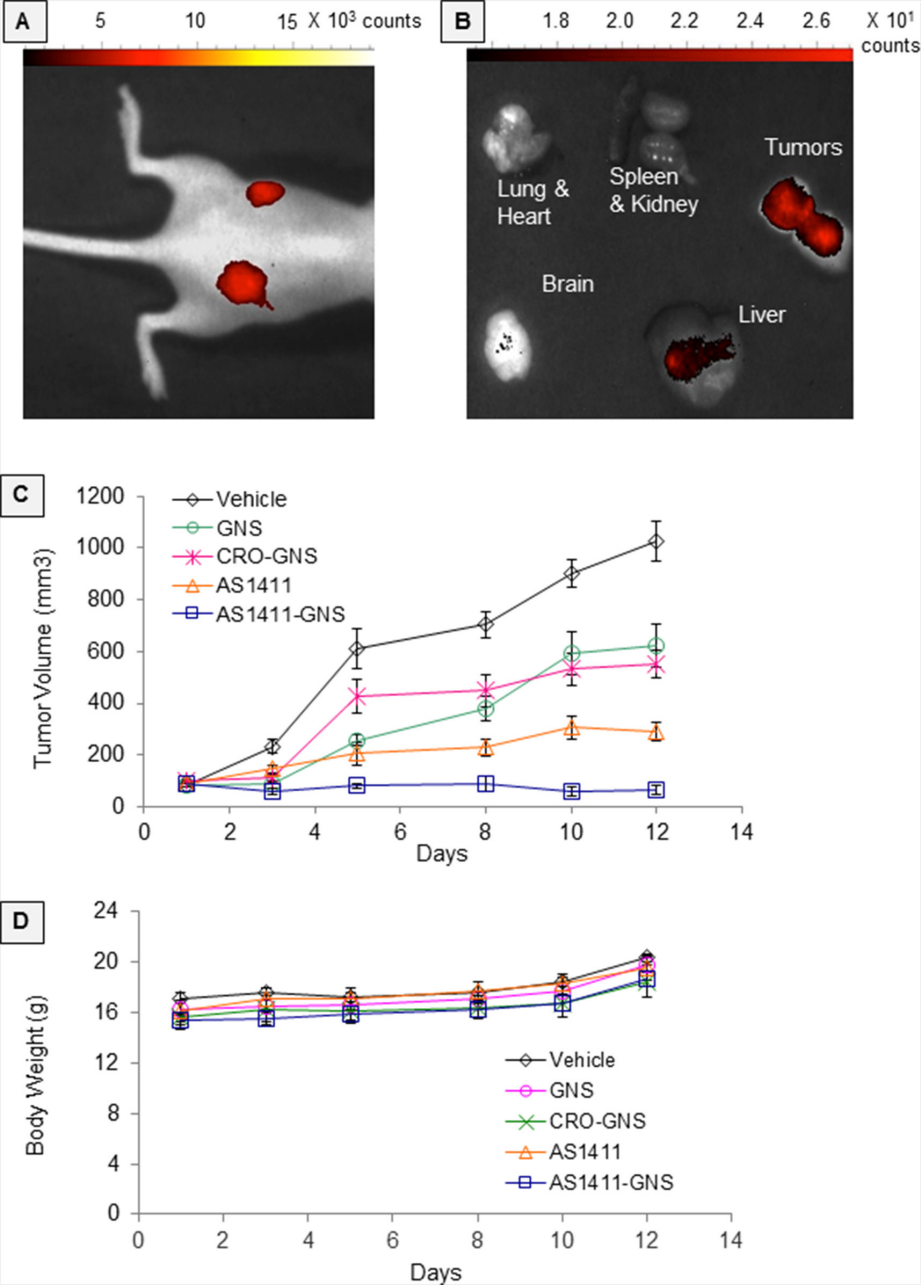
*In vivo* studies Experiments in female nude mice bearing MDA-MB-231 xenografts on both flanks: **A.** *In vivo* fluorescence imaging at 7 days after a single intravenous administration (retro-orbital) of Cy5-AS1411-GNS or Cy5-CRO-GNS. **B.** Fluorescence imaging of resected organs at 6 days after intravenous administration (tail vein) of Cy5-AS1411-GNS. **C.** Antitumor efficacy studies. When tumors were ~100 mm^3^, mice were randomly assigned to five groups (5 mice/10 tumors per group) to receive daily injections as indicated for 12 days. Tumor volumes were calculated from caliper measurements on the days indicated. The graph shows mean calculated tumor volume and standard error. **D.** Mean body weights and standard error for the groups of mice shown in panel C.

Before initiating *in vivo* efficacy studies, we performed preliminary biodistribution studies in order to verify the bioavailability of AS1411-GNS. Following systemic administration of Cy5-labeled AS1411-GNS in nude mice bearing MDA-MB-231 xenograft tumors, tumor-selective accumulation of fluorescence was observed by *in vivo* fluorescence imaging of live mice and by fluorescence imaging of organs *post-mortem*, as shown in Figure 4A and 4B. Accumulation of AS1411-GNS in the tumor could be observed in live mice at the earliest time point examined (2 h, data not shown) and persisted until at least 6–7 days, by which time there was minimal fluorescence observed in normal organs (Figure 4A and 4B). These data suggest that the AS1411-GNS are selectively taken up and retained in the tumor but cleared from normal tissues. Although more extensive quantitative studies of AS1411-GNS biodistribution and pharmacokinetics will be needed to fully define the optimal dose, schedule, and administration route, the results shown provided evidence of bioavailability of AS1411-GNS and encouraged us to proceed to proof-of-concept antitumor efficacy studies. For these, nude mice with subcutaneous MDA-MB-231 xenografts received daily injections for 12 consecutive days with 22 μg oligonucleotide per day (approximately 1 mg/kg/day) of AS1411-GNS, CRO-GNS, or AS1411. Additional control groups received an equivalent amount of unconjugated GNS or vehicle alone. Tumor volume and body weight were recorded at regular intervals. The results (Figure 4C) indicate that AS1411-GNS can completely inhibit the tumor growth and induce tumor regression in this model. The AS1411-GNS treatment was significantly more effective than the equivalent dose of AS1411, CRO-GNS, or GNS (*p* < 0.05). Furthermore, there was no evidence of any toxicity for any of the treatments, as judged by body weight (Figure 4D), behavior of the mice, or examination of organs.

## DISCUSSION

The major goal of this study was to test the prediction that attaching an anticancer aptamer, AS1411, to GNS would improve its antitumor efficacy in an animal model of cancer and the results we have obtained so far (Figure 4C) support this hypothesis. Our data from experiments performed using cultured breast cancer cells indicate that the AS1411-GNS conjugates display superior potency—including higher cellular internalization, improved growth inhibitory effects, and increased cytotoxicity—relative to AS1411 alone or to control GNS conjugates, while still maintaining selectivity for cancer cells over non-malignant cells (Figures 2 and 3). These enhancements in AS1411 biological activities most likely contribute to the observed improvement in antitumor efficacy for AS1411-GNS compared to AS1411, although it appears that the GNS themselves may have some inherent activity in this model as the GNS and CRO-GNS have a modest antitumor effect (Figure 4C). Enhanced accumulation in tumors may also play a role and our preliminary *in vivo* investigations are suggestive of this (Figures 4A and 4B), but more detailed biodistribution and pharmacokinetics studies will be required to confirm this and we plan to pursue these in the future.

Another priority for future studies will be to elucidate the mechanism of action for AS1411-GNS. Our group and a number of independent research teams have previously demonstrated that several biological activities of AS1411 are dependent on nucleolin (e.g. by showing that nucleolin siRNA inhibits AS1411 effects) [2, 9, 10, 15, 17, 18], so it is probable that the AS1411-GNS behave similarly. However, even though a role for nucleolin in AS1411 activity has been established and the differential distribution of nucleolin in malignant cells provides a rationale for cancer-selectivity, it remains unclear exactly how binding of AS1411 to nucleolin leads to efficient cellular internalization in cancer cells and ultimately to cell death. A very recent paper from the Mirkin group reported that when AS1411-containing oligodeoxynucleotides were attafched to GNS, G-quadruplex formation was enhanced and binding to selected serum proteins was increased relative to AS1411 [19], which may also be relevant to the mechanism.

Regardless of the mechanistic details, the work presented in this paper adds to the growing body of evidence that attaching AS1411 is an excellent strategy for delivering a wide variety of nanoparticles or other entities to the inside of cancer cells (see [20–29] for examples). Furthermore, our studies are broadly consistent with the work of Odom and colleagues, who have extensively characterized AS1411-linked gold nanostars and their potential for cancer therapy [30–33]. Similar to the AS1411-GNS described in this paper, the AS1411-linked gold nanostars have efficient uptake in cancer cells and potently induce cell death in cancer cells but not in non-malignant cells [30–33]. This group also very recently described the *in vivo* toxicology and biodistribution of the AS1411-linked gold nanostars; they found that the nanoconstruct had no evidence of toxicity and showed substantial tumor accumulation in a breast cancer xenograft model similar to that used here [34].

Detailed *in vitro* and *in vivo* comparisons between comparable nanosphere and nanostar AS1411-linked constructs will be required in the future to determine which of these could be most useful for cancer therapy. The greater surface area of nanostars relative to nanospheres allows increased loading of AS1411, but the AS1411-GNS may have advantages over AS1411-linked gold nanostars in other respects. Nanostars are inherently non-uniform in terms of shape, which may have consequences for both the structural stability of AS1411-nanostars and how they interact with biomolecules and membranes. Exchange rates with endogenous thiols for individual aptamers bound to a nanostar may vary widely due to the sharp changes in surface curvature from positive to negative. As exchange rates tend to increase with curvature, they may be higher for nanostars compared to nanospheres. Also, the non-spherical nature of nanostars may be more likely to invoke immune responses and cellular damage. Nanospheres avoid these potential complications because they are monodisperse in size and shape and have uniform curvature. This will lead to uniform coatings of aptamer that are resistant to thiol exchange and will have consistent interactions with biomolecules. Likewise, one batch of nanospheres should be consistent with the next, and display stable and predictable pharmacokinetics.

In summary, our *in vivo* work has provided proof-of-principle data demonstrating the antitumor efficacy of AS1411-GNS in a mouse model of breast cancer, while our cell-based studies have added to the expansive data supporting the ability of AS1411 to deliver cargoes efficiently and selectively to the inside of cancer cells. The results reported herein establish the potential of AS1411-GNS as a future nanomedicine and highlight the need for additional research—especially *in vivo* studies—to facilitate translation to the clinic.

## MATERIALS AND METHODS

### Cell culture

The cell lines, MCF-7, MDA-MB-231, and MCF10A, were obtained from American Type Culture Collection (ATCC). MCF-7 and MDA-MB-231 cells were maintained in DMEM growth medium with 10% fetal bovine serum (FBS, Hyclone) and 100 IU/mL penicillin-streptomycin (Invitrogen). For MCF-7 only, 0.01 mg/mL human recombinant insulin (Sigma) was also added. MCF10A were maintained in MEBM basal medium supplemented with MEGM SingleQuots (Lonza). All cell lines were maintained at 37°C under 5% humidified CO_2_.

### Preparation of oligonucleotide-conjugated gold nanospheres

Colloidal gold nanospheres (GNS, 5 nm diameter) were purchased from Ted Pella Inc. (Redding, CA) or Nanopartz (Loveland, CO). Oligonucleotides were purchased from Integrated DNA Technologies (San Diego, CA). All oligonucleotides had a regular DNA backbone (i.e. phosphodiester), a 5′-Thiol C6 S-S modification (Thio-MC6-D), and were HPLC purified; those used for imaging studies also had a 3′-Cy5 fluorophore modification. Oligonucleotides sequences consisted of AS1411 (nucleolin aptamer, 5′-GGTGGTGGTGGTTGTGGTGGTGGTGG) or CRO control (5′-CCTCCTCCTCCTTCTCCTCCT CCTCCT) flanked by 5′-T_6_ and 3′-T_3_ spacer sequences (the latter to reduce fluorescence quenching). Oligonucleotides were dissolved in nuclease/endotoxin-free 18.2 MΩ water (Invitrogen) to give a stock solution concentration of 500 μM. All other solutions were prepared using nuclease/endotoxin-free water and then filtered, and all mixing was performed under the laminar flow hood to prevent any contamination. Conjugation of oligonucleotides to gold nanospheres was achieved using a method similar to those described in Javier *et al*. [35], based on the original protocol of Mirkin *et al*. [36]. Thiolated oligonucleotides were first reduced by incubating with 10 mM tris-(2-carboxyethyl)-phosphine hydrochloride (TCEP, Pierce) for 30 min at room temperature followed by desalting using an NAP-5 filter column (Sephadex G-25, GE Healthcare), and then were added to 10 mL of 5 nm colloidal gold nanospheres (1.4 × 10^14^ nanospheres/mL) to give a final oligonucleotide concentration of 50 μM (3.0 × 10^16^ oligonucleotides/mL). After overnight incubation on a rotator and/or occasional sonication, particles were aged slowly at room temperature by adding 10X phosphate buffered saline (PBS, Sigma Aldrich, St. Louis, MO) every 12 h over a period of 24–48 h until 1X concentration was reached. Following incubation, the gold nanoparticle complexes were divided into ten 1.5 mL tubes (LoBind, Eppendorf) and centrifuged at 13,500 *g* for 30 minutes to separate the unconjugated oligonucleotide from the complex. The pellet was washed with 1X PBS and centrifuged again for 30 min. The sedimented gold nanoparticle complexes were reconstituted in 1X PBS and pooled in a single tube to give 1 mL final volume. To determine the concentration of oligonucleotide attached to the gold nanospheres (GNS), aliquots of the oligonucleotide-GNS complexes were incubated for 2 h at room temperature with 10 mM dithiothreitol (DTT, Sigma Aldrich, MO) to release the bound oligonucleotide. After centrifugation at 13,500 *g* for 30 min, the supernatant was collected and released oligonucleotides were quantified using Nanodrop spectrometer (Thermo Scientific). To sterilize, the gold nanoparticle constructs were poured into a sterile 35 mm dish and exposed to ozone (Cool Clave) before use. The product was stored at 4°C in amber colored 1.5 mL tubes (Eppendorf) until further use.

### Physical characterizations

Hydrodynamic size and zeta potential analyses were performed using Brookhaven Instruments Particle Size Analyzer (90Plus) or Malvern Zetasizer Nano-ZS90 with 633 nm He-Ne laser (Malvern Instruments, Inc., Westborough, MA). Particles were diluted 1:5 in 1 mL of distilled water for these measurements. Scanning transmission electron microscopy (STEM) was performed using a Zeiss Supra 35 (Carl Zeiss Microscopy, LLC, Thornwood, NY) field emission scanning electron microscope with STEM detector. Samples were prepared by placing 10 μL of particles dispersed in DI water on Formvar/Carbon 200 mesh copper grids and allowing the samples to dry overnight. Images were captured using EHT = 20 kV. To determine whether particles aggregated in various solvents or in the presence of serum (FBS) or in complete cell culture media (RPMI+10% FBS), 500 μL aliquots of conjugated particles (OD 0.5) were incubated at room temperature with 2 mL of solvent for 5–7 days (as indicated in figure legends) and the color was recorded. Red/pink color was considered indicative of well-dispersed particles, whereas purple/blue to gray/colorless was considered to indicate increasing degrees of aggregation.

### Cellular uptake studies

To study nanoparticle uptake in breast cancer cells (MDA-MB-231 and MCF-7) and non-malignant cells (MCF10A breast epithelial cells), we utilized oligonucleotide-GNS complexes that were synthesized using oligonucleotides with a 3′-fluorophore (Cy5) modification. For fluorescence microscopy, an Olympus CKX41 fluorescence microscope attached with Q Color5 CCD camera and Q Capture Pro 6.0 imaging software were used. Cells were seeded onto chambered glass slide and incubated with various nanospheres or controls in a humidified incubator with 5% CO_2_ at 37°C. Following incubation, cells were washed twice with Hanks Balance Salt Solution (HBSS) and fixed with 4% paraformaldehyde in PBS, stained with DAPI or Hoechst 33342 (nuclear stain) and/or with wheat agglutinin (WGA)-Alexa 488 conjugate (plasma membrane stain), as per the instructions from the manufacturer (Molecular Probes). For confocal microscopy, 10^3^ cells were seeded onto 35 mm FluordishB plates (World Precision Instrument) and incubated in a humidified incubator at 37°C with 5% CO_2_ for 16 h. Cells were stained as described above and images were taken on Nikon A1 confocal system (Nikon USA). NIS Elements Image software was used for acquisition. For silver-enhanced staining to visualize gold nanospheres, we used the Silver Enhancement Kit (Ted Pella Inc.). Cells were seeded onto four-well chamber slides (Millipore) and incubated 24 h, then the medium was changed and cells were treated with various GNS-containing complexes. After 48–72 h, cells were washed twice with PBS (Invitrogen) and fixed with 4% paraformaldehyde for 30 min. Following fixation, cells were washed three times with PBS and once with deionized water, then slides were allowed to air dry. Three drops of initiator (Solution A) were mixed with three drop of enhancer (Solution B) in an Eppendorf tube, then applied drop wise onto the slide for 5 min. The slides were then washed several times with deionized water and mounted with Mounting Solution (Invitrogen) and cover slips, before viewing under a light microscope.

### *In vitro* assays for cell proliferation, clonogenic survival, and cell death

These assays were performed using previously published protocols [16, 37] and are described briefly here. For cell proliferation assays, 10^3^ cells/well were seeded in 96 well plates in triplicate and incubated overnight to allow adherence. Cells were then treated by addition of oligonucleotide-GNS conjugates, or an equivalent amount of GNS alone, to the cell culture medium to give the final concentrations indicated. After 72 h, cell proliferation was determined using the colorimetric MTT (3-[4, 5-dimethylthiazol-2-yl]-2, 5-diphenyltetrazolium bromide) assay. Plates were read at 570 nm using a plate reader (Synergy HD, BioTek, VA, USA). To control for interference of gold nanospheres in the assay, we subtracted the background value for the culture medium with gold nanospheres. For the clonogenic survival assays, cells were harvested in single cell suspension and 10^3^ cell/well were seeded in six well plates. After 24 h of incubation at 37°C, cells were treated with different concentrations of oligonucleotides or oligonucleotide-conjugated GNS or an equivalent amount of GNS alone, then incubated for a further 10 days. Cells were then fixed with 4% paraformaldehyde, stained with Cell Stain (Cell Signaling, USA), and colonies with ≥ 50 cells were counted. To evaluate cell death, cells (5 × 10^4^) were plated in a T-25 flask in complete medium and treated as indicated. After 72 h treatment, cell death was determined by flow cytometric analyses (with Flow Jo software ver. 7.6.5) of cells stained with FITC Annexin V and propidium iodide (PI) using the Apoptosis Detection Kit (BD Bioscience).

### *In vivo* studies

The Institutional Animal Care and Use Committee (IACUC) at the University of Louisville approved all experimental procedures involving animals. Healthy female weanling nude mice (Fox1^nu^), were purchased from Harlan Inc, (Indianapolis, IN). After acclimation for a week in the animal facility, mice were injected subcutaneously with a single cell suspension consisting of 10^7^ MDA-MB-231 cells in 200 μL of PBS into each flank. When the subcutaneous tumors reached the volume of approximately 100 mm^3^, the mice were randomized into five groups of 5 mice per group (10 tumors per group) and treated by daily intraperitoneal injection for 12 consecutive days with 100 μL of a sterile PBS solution containing one of the following: 22 μg AS1411-GNS, 22 μg CRO-GNS, an equivalent amount of unconjugated GNS, 22 μg AS1411 (without GNS), or vehicle alone (PBS). Tumor volume and body weight were recorded after every 2 or 3 days for 12 days. To examine biodistribution in this animal model, oligonucleotide-GNS complexes that had been synthesized using oligonucleotides with a 3′-fluorophore (Cy5) modification were administered. Tumor uptake was then visualized by fluorescence imaging of live animals or of resected organs following euthanasia.

### Statistical analyses

Unless otherwise stated, quantitative results were expressed as the mean ± SEM of at least three independent experiments. Statistical significance of the studies was evaluated by using One-way ANOVA and *p* values ≤ 0.05 were considered significant. All data was analyzed using GraphPad Prism version 6.0, GraphPad Software, La Jolla California USA.
